# GenAI-Supported Flipped Learning in Preservice Chemistry Teacher Education: Lesson-Design Performance, Learning Attitude, Self-Regulated Learning, and Critical Thinking Awareness

**DOI:** 10.3390/bs16040514

**Published:** 2026-03-29

**Authors:** Jun Zhang, Xinyue Deng, Tong Wu, Kai Wang

**Affiliations:** 1 College of Teacher Education, Southwest University, Chongqing 400715, China; zhangyi1231@swu.edu.cn (J.Z.);; 2 Center for Teacher Education Research, Beijing Normal University, Beijing 100875, China

**Keywords:** generative artificial intelligence, flipped learning, preservice chemistry teachers, lesson-design performance, learning attitude, self-regulated learning, critical thinking awareness

## Abstract

This quasi-experimental study compared GenAI-supported flipped learning (AI-FL) with reading-based flipped learning (R-FL) in an 11-week preservice chemistry course. Two intact classes completed the same topics and identical in-class activities, differing only in pre-class preparation through guided GenAI-based interactive learning or assigned readings. The study examined lesson-design performance, learning attitude, self-regulated learning, and critical thinking awareness. After controlling for pretest scores, the reading-based flipped learning group showed stronger lesson-design performance, whereas the GenAI-supported group reported more positive learning attitudes. No significant group differences were observed for self-regulated learning or critical thinking awareness. These findings suggest that, in this course context, GenAI-supported pre-class learning may enhance learners’ attitudes but does not necessarily improve rubric-aligned lesson-design performance compared with reading-based preparation.

## 1. Introduction

In recent years, the rapid diffusion of generative artificial intelligence (GenAI) in higher education has made it increasingly common for university students, including preservice teachers, to rely on tools such as ChatGPT (GPT-4) for information search, brainstorming, and writing support (Chan & Hu, 2023; Ravšelj et al., 2025). In parallel, flipped learning (FL)—a blended approach that emphasises self-directed preparation before class and interactive deepening of learning during class—has been widely adopted in higher education. Syntheses of empirical evidence suggest that FL can, overall, improve students’ learning performance and engagement (Akçayır & Akçayır, 2018; Van Alten et al., 2019).

However, the pre-class phase of traditional FL often suffers from unstable preparation and fluctuating participation. Because out-of-class learning typically lacks timely guidance and opportunities for question resolution, students may develop conceptual gaps that amplify individual differences in learning progress (Baig & Yadegaridehkordi, 2023; Z. Sun & Xie, 2020). Accordingly, the effectiveness of FL depends substantially on whether learners can enact self-regulated learning (SRL) effectively during pre-class study (Shih et al., 2019).

GenAI introduces new possibilities for strengthening the pre-class component of FL. However, empirical evidence remains mixed regarding whether GenAI functions as an effective scaffold that promotes deeper learning, or as a convenience tool that encourages shallow processing and overreliance. Prior research suggests that GenAI can enhance motivation, self-efficacy, and engagement by providing on-demand explanations and personalised feedback (Xia et al., 2025; Yilmaz & Karaoglan Yilmaz, 2023), and may support goal setting and task investment in flipped learning environments (W. Huang et al., 2019). At the same time, experimental evidence suggests that the convenience of GenAI can foster overreliance, weakening evaluation and self-monitoring and potentially limiting deeper information integration (Barcaui, 2025; Chen et al., 2025).

These tensions are particularly salient in teacher education, where preservice teachers are increasingly expected to use GenAI to support lesson planning and pedagogical decision-making, yet may also be vulnerable to uncritical adoption of AI-generated suggestions. From a constructivist perspective, what matters is not merely access to support but whether scaffolds promote active meaning making and sustained monitoring within authentic tasks. Against this backdrop, the present study implemented an 11-week quasi-experiment in an authentic course context to examine how GenAI may be integrated into flipped learning. Specifically, we compared GenAI-supported flipped learning (AI-FL) with reading-based flipped learning in terms of rubric-scored lesson-design performance, learning attitude, self-regulated learning, and critical thinking awareness. Overall, the findings suggest that GenAI-supported pre-class learning was associated with more positive learning attitudes, whereas reading-based preparation yielded stronger rubric-aligned lesson-design performance; no statistically significant between-group differences were observed for SRL or critical thinking awareness.

## 2. Literature Review

### 2.1. Constructivism and Flipped Learning

Constructivist learning theory, informed by Piaget and Vygotsky, provides a theoretical lens for explaining the pedagogical logic of flipped learning (FL). From a constructivist standpoint, learning is not the passive reception of information but an active process of meaning-making through experience, reflection, and social interaction. Piaget highlighted cognitive development through assimilation and accommodation, whereas Vygotsky emphasised mediation by cultural tools and support within the Zone of Proximal Development (ZPD) (Arbaugh et al., 2009; Xue, 2023). These perspectives converge on the idea that learning is strengthened when learners are actively engaged and supported through guidance and dialogue (Pricopie, 2020). FL operationalises these principles by shifting initial content engagement to the pre-class phase and reserving class time for interaction, application, and feedback. Bishop and Verleger (2013) characterised FL as combining computer-mediated individual learning prior to class with interactive group-based activities during class. In this design, knowledge construction is initiated through autonomous pre-class exploration and deepened via social co-construction in the classroom, consistent with constructivist emphases on learner agency and collaborative meaning negotiation (Rob & Rob, 2018). Empirical studies and reviews suggest that FL can enhance learning outcomes and engagement, particularly when in-class time is used for active learning rather than content transmission (C.-L. Lai & Hwang, 2016; Smith, 2013), aligning with broader evidence on the efficacy of active learning strategies (Prince, 2004). However, FL is not automatically effective. Motivational accounts suggest that FL may support autonomous motivation by increasing opportunities for autonomy, competence, and relatedness (Ryan & Deci, 2000). At the same time, insufficient scaffolding during pre-class preparation can increase cognitive burden and frustration, undermining participation and subsequent in-class learning (Abeysekera & Dawson, 2015). Jensen et al. (2015) further argued that the effectiveness of flipped instruction depends less on sequence reversal itself than on whether learners are supported to engage in active, constructivist-aligned learning activities. Accordingly, FL should be understood as a framework whose outcomes hinge on the quality of pre-class scaffolding and the extent to which learners can regulate and monitor their preparation effectively and reflectively. As digital tools evolve, they may offer new forms of pre-class support and feedback that reshape how scaffolding is provided in FL settings (Carby, 2023). This development provides a rationale for examining GenAI as a pre-class scaffold in a flipped lesson-design course for preservice teachers.

### 2.2. Generative AI in Higher Education

In higher education, GenAI has been increasingly discussed as an on-demand scaffold that can support students’ out-of-class learning. Reviews suggest that GenAI tools can assist with information search, drafting, concept explanation, and formative feedback across contexts such as academic writing and STEM learning (Kasneci et al., 2023; Zawacki-Richter et al., 2019). These affordances are particularly relevant to flipped learning, where the quality of pre-class preparation hinges on learners’ access to timely guidance during self-directed study.

Meta-analytic evidence on GenAI-supported instruction suggests overall small-to-moderate positive effects on students’ academic performance, along with modest positive influences on motivational outcomes such as engagement and interest (Deng et al., 2025; Liu et al., 2025; Xia et al., 2025). At the same time, empirical studies show that well-designed GenAI learning environments can foster learning motivation and, when embedded in structured learning tasks, support self-regulated learning processes by prompting learners to plan, monitor, and reflect on their work (Bai & Wang, 2025; Gao et al., 2024; Shi et al., 2025; Trinovita et al., 2025). In such designs, GenAI functions not merely as an answer generator but as a tool that can externalize and guide strategic learning processes.

In science and STEM education, studies report that GenAI can help students make sense of abstract concepts, engage in modelling and inquiry activities, and receive structured feedback on scientific problem solving and written products, including in chemistry-related courses (Bewersdorff et al., 2025; Zhai et al., 2025).

However, a growing body of research also cautions that GenAI may induce cognitive offloading. Although GenAI-supported inquiry can reduce students’ perceived cognitive load, it may simultaneously weaken the depth and quality of reasoning and argumentation relative to more traditional information-search approaches, especially when learners over-rely on AI-generated suggestions (Fan et al., 2025; Tian & Zhang, 2025). Survey and process data further indicate that stronger reliance on AI tools is often associated with lower levels of critical thinking, and that deep processing and durable learning may be undermined when students depend heavily on GenAI outputs. While GenAI can expand access to personalised and timely support, when used without sufficient reflection and regulation it may erode learners’ metacognitive engagement and their capacity to critically evaluate AI-generated content.

### 2.3. AI-Supported Flipped Learning

AI-supported flipped learning (AI-FL) has been proposed as a way to address limitations of traditional flipped classrooms, particularly the lack of timely feedback and limited individualised support during the pre-class preparation phase (Lo & Hew, 2023). Here, AI-FL is used as an umbrella term; however, the present study examines its GenAI-supported form. Early implementations often relied on rule-based or retrieval-based chatbots whose functions were confined to answering frequently asked questions, providing learning navigation, or offering simple prompts and examples (Diwanji et al., 2018; W. Huang et al., 2019). As a result, such systems provided only limited support for complex learning processes. With the development of generative artificial intelligence (GenAI), AI-FL has gained more advanced language-generation and feedback capabilities, enabling more interactive explanations and guidance during pre-class learning.

At the same time, GenAI-generated responses may appear fluent yet contain inaccuracies or unsupported claims. Accordingly, teachers may need to monitor and calibrate AI-mediated guidance, which can in some cases increase rather than reduce workload (Selwyn, 2024). Recent empirical studies suggest that embedding GenAI into flipped learning designs can enhance learning performance, motivation, and metacognitive outcomes, particularly when use is integrated with structured prompts, task requirements, and checkpoints (Li, 2023; Namaziandost, 2025). However, other research cautions that some students may develop a pattern of obtaining answers quickly while engaging in less self-processing. Lower-achieving students may be especially prone to overreliance on GenAI outputs, which can undermine deep learning and knowledge internalisation (Tian & Zhang, 2025).

### 2.4. Learner-Related Variables in GenAI-Supported Flipped Learning

The present study focuses on three subjective, student-centred variables: learning attitude, self-regulated learning (SRL), and critical thinking awareness. These constructs reflect learners’ motivational orientations, their capacity to proactively regulate learning processes, and their tendency to critically evaluate information.

In this study, learning attitude refers to students’ overall evaluative stance toward the course and its learning activities. It includes perceived course value, interest in learning, and willingness to invest effort. Closely related to learning motivation, learning attitude shapes the depth and persistence of students’ engagement in instructional activities (Collie & Martin, 2019). It helps explain why, and to what extent, students devote effort to pre-class preparation and in-class tasks. Prior research also indicates that learning attitude is reciprocally related to learning and achievement over time (C. Huang, 2011; Vu et al., 2024). From a self-determination theory perspective, more autonomous and self-endorsed motivation is more likely to foster deep learning and sustained engagement (Ryan & Deci, 2000).

Self-regulated learning (SRL) is typically defined as a constructive process in which learners actively regulate their thoughts, motivation, and behaviours to attain learning goals (Zimmerman, 2001, 2002). Zimmerman’s cyclical model differentiates three interrelated phases—forethought, performance, and self-reflection. Together, these phases describe how learners set goals, plan and implement strategies, monitor progress, and evaluate outcomes (Panadero, 2017). SRL has been consistently identified as a key precondition for benefiting from flipped classroom designs because substantial knowledge construction is shifted to pre-class self-study and individual responsibility (Shih et al., 2019; Sletten, 2017; Van Alten et al., 2020). However, studies suggest that many preservice teachers show weak goal setting during pre-class preparation and seldom apply monitoring strategies when studying at home. This pattern can lead to superficial preparation and fragmented understanding of course content (Brewer & Movahedazarhouligh, 2018; Çakıroğlu & Öztürk, 2017; Zheng et al., 2020). Overall, SRL appears to be a key condition for effective learning in flipped classrooms (C.-L. Lai & Hwang, 2016).

Critical thinking is commonly defined as purposeful, self-regulatory judgment involving analysis, evaluation, inference, and reflection (Abrami et al., 2015; Facione, 2015; E. R. Lai, 2011). In educational research, critical thinking is discussed both as a set of reasoning skills and as a dispositional tendency to apply those skills in learning situations (Ennis, 1985). As the present study uses a self-report measure, it does not directly assess critical thinking skills. Instead, it examines critical thinking awareness, defined as learners’ perceived propensity to monitor and evaluate information and evidence during learning activities.

Taken together, these three learner-related variables are theoretically relevant to the pre-class phase of flipped learning, where students must sustain motivation, regulate their preparation, and evaluate information sources. Accordingly, these constructs provide a theoretically grounded set of learner-related outcomes for contrasting GenAI-supported and reading-based pre-class learning conditions.

## 3. Research Objectives, Research Questions, and Hypotheses

Despite growing interest in GenAI-supported learning in teacher education, evidence remains limited regarding its impact on preservice teachers’ learning outcomes and learner perceptions in subject-specific pedagogy courses. Recent research on STEM teacher preparation has highlighted the growing importance of technology integration and the need for more context-specific empirical evidence in pedagogical settings (Rehman et al., 2025). In particular, few studies have simultaneously examined both objective learning performance and subjective perceptions in GenAI-supported flipped learning within chemistry lesson-design contexts. To address these gaps, the present study uses the Chemistry Lesson Design course as the research context and examines the effects of GenAI-supported flipped learning on preservice teachers’ learning outcomes and perceptions. Accordingly, the following research questions and hypotheses were proposed:

**RQ1.**  *Compared with reading-based flipped learning, does GenAI-supported flipped learning influence preservice teachers’ objective lesson-design performance in chemistry?*

**H1.**  *GenAI-supported flipped learning will lead to significantly different lesson-design performance from reading-based flipped learning.*

**RQ2.**  *Compared with reading-based flipped learning, does GenAI-supported flipped learning influence preservice teachers’ subjective learning perceptions (i.e., learning attitude, self-regulated learning, and critical thinking awareness)?*

**H2.**  *GenAI-supported flipped learning will lead to significantly different subjective learning perceptions from reading-based flipped learning.*

## 4. Materials and Methods

### 4.1. Research Design

This study adopted a quasi-experimental design, specifically a pretest–posttest nonequivalent-groups design, to examine the effects of two flipped learning approaches on preservice teachers’ learning outcomes and learner-related variables. Because random assignment was not feasible in the natural classroom context, two intact classes enrolled in the same Chemistry Lesson Design course were selected as the sample. One intact class was designated as the experimental group (AI-FL), while the other served as the control group (R-FL). Group assignment followed the existing class arrangement (intact classes) rather than individual randomization. Therefore, potential class-level effects cannot be fully ruled out, and the observed intervention effect cannot be cleanly separated from pre-existing differences between the two classes.

The study was conducted over an 11-week course schedule, with the instructional treatment implemented through nine guided-question sessions. Pretest measures were collected at the beginning of the study, whereas posttest measures and the final lesson-design task were completed at the end of the study. Pretest scores were used to examine baseline equivalence and were entered as covariates in subsequent analyses to strengthen the validity of between-group comparisons.

### 4.2. Participants and Context

A total of 53 preservice chemistry teachers were recruited from a comprehensive university. Participants were drawn from two intact classes enrolled in the same course, Chemistry Lesson Design (experimental group: *n* = 24; control group: *n* = 29). All participants were second-year undergraduate students majoring in chemistry teacher education. In terms of gender, 33 participants were female (62%) and 20 were male (38%). Although specific age data were not collected in the questionnaire, all participants were from the same year level and program and were therefore of comparable age. Prior to the intervention, participants had not received systematic instruction in GenAI-supported flipped learning, although some may have had limited prior exposure to flipped classroom activities or GenAI tools. All participants voluntarily took part in the study and provided informed consent prior to data collection. The course was taught by the same instructor for both classes. The syllabus, weekly topics, in-class activities, timeline, and assessment criteria were kept identical across conditions.

### 4.3. Intervention and Procedure

**Figure 1:**
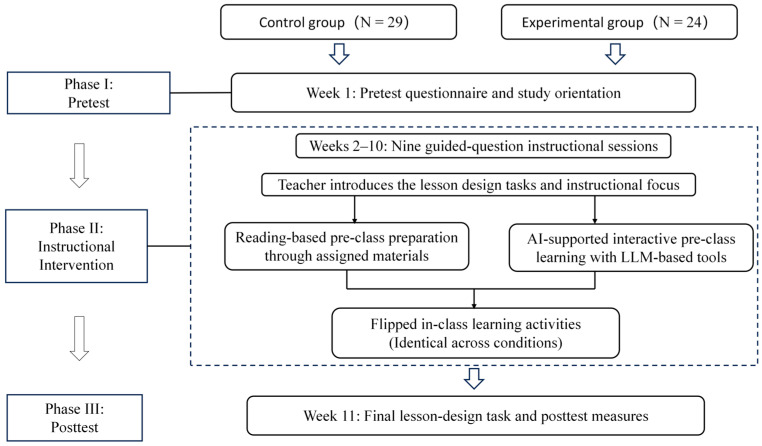
Study timeline and procedure of the quasi-experimental intervention.

The instructional intervention was implemented within the course Chemistry Lesson Design, offered during the spring semester of 2025 and spanning 11 weeks. As illustrated in Figure 1, Week 1 was used for the pretest questionnaire and study orientation, Weeks 2–10 comprised nine guided-question instructional sessions, and Week 11 was used for the final lesson-design task and posttest measures. During the Week 1 study orientation, the instructor explained the purpose and procedure of the quasi-experimental study, obtained informed consent from all participants, and clarified the different pre-class learning requirements for the two conditions. Students in the R-FL condition were explicitly informed that their pre-class preparation should be completed through the assigned reading materials rather than through AI-based tools. The course aimed to develop preservice teachers’ competencies in chemistry lesson design, covering instructional objectives, learning contexts, teaching methods, learning activities, and assessment strategies. The intervention consisted of two phases: a pre-class learning phase and an in-class activity phase. Across the nine instructional sessions, both instructional conditions addressed identical instructional topics and lesson design tasks, differing only in the format and interaction mode of pre-class learning. The overall intervention procedure and the two instructional conditions are summarised in Figure 1. Pre-class preparation in both conditions was structured around the same instructor-provided guiding-question scaffold, consisting of the upcoming lesson topic and three to four core guiding questions. An overview of the weekly topics and instructor-provided guiding questions used during the intervention is provided in Appendix A.

**Figure 2:**
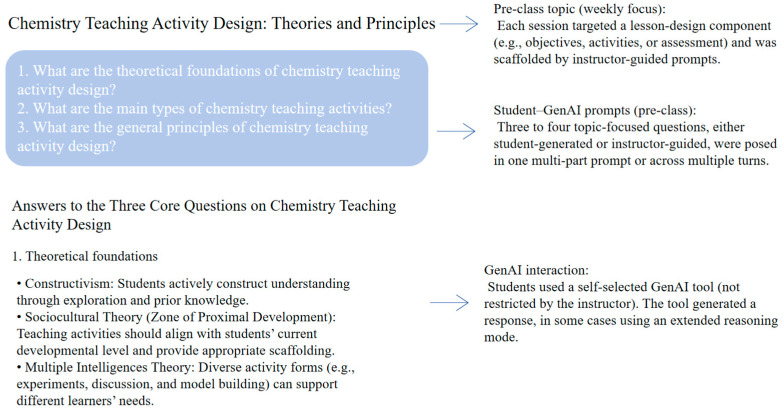
Schematic of preservice teachers’ pre-class GenAI interaction in the AI-FL condition.

During Weeks 2–10, students completed weekly pre-class preparation for lesson-design tasks assigned by the instructor. In both conditions, the instructor first announced the topic of the upcoming lesson and provided three to four core guiding questions to direct students’ preparation. In the AI-FL condition, students engaged in interactive pre-class learning using large language model–based tools. A schematic illustration of this pre-class GenAI interaction process is presented in Figure 2. The instructor provided brief guidance on how to ask task-related questions and refine follow-up queries, and students used GenAI tools to explore the topic and develop responses to the guiding questions. In the R-FL condition, students completed pre-class preparation through assigned readings on the same instructional topics. The instructor provided relevant reading materials, including curriculum standards, instructional design literature, and exemplary lesson cases. Students were required to read and synthesise these materials and prepare responses to the same guiding questions without AI-based support. Before each weekly pre-class assignment, the instructor reiterated this requirement to reinforce adherence to the group-specific learning procedure.

To monitor implementation fidelity, students in both conditions were required to submit records of task completion after each of the nine guided-question instructional sessions. These submissions documented completion of the corresponding pre-class learning tasks. In the AI-FL group, students submitted screenshots of their AI interactions together with responses to the guiding questions. In the R-FL group, students submitted written responses to the same guiding questions based on the assigned reading materials. For the AI-FL group, the submitted screenshots were required to display the guiding questions, students’ responses, and key AI dialogue turns. These materials were used solely to verify task completion and monitor students’ engagement with the assigned pre-class tasks; they were not included in grading or outcome scoring. Students anonymised personal identifiers prior to submission, and all files were stored securely for research purposes only.

During the in-class phase, instructional activities were identical across the two conditions. Classroom activities focused on group discussions and case analyses related to weekly themes. Students compared alternative lesson design approaches, identified key design principles, and completed corresponding in-class tasks. Throughout the course, students progressively applied these principles to subsequent lesson-design practices, culminating in a comprehensive lesson-design assignment completed in Week 11.

### 4.4. Measures

#### 4.4.1. Objective Performance Task: Lesson Design Performance

In the present study, preservice teachers’ lesson-design performance was used as a rubric-scored performance outcome. Lesson-design products were evaluated using a standardized scoring rubric adapted from the official evaluation criteria of the Tian Jiabing Cup National Teaching Skills Competition, a nationally recognized competition for preservice teachers in China. The rubric assesses lesson-design quality across multiple dimensions, including objective design, content analysis, learner analysis, teaching process design, extension design, document quality, and design innovation. The original rubric yields a total score of 25 points, and the full original rubric is provided in Appendix B (Table A2).

To align the rubric with the course assessment system, the original 25-point scale was linearly rescaled to a 100-point scale. This transformation adjusted only the total score range and did not change the relative weighting or substantive meaning of any evaluation dimension, thereby preserving the conceptual integrity and internal structure of the rubric.

All lesson-design products were independently scored by two trained raters using the same standardized rubric for both pretest and posttest products. Prior to formal scoring, the raters received training and calibration using sample products. Inter-rater reliability for the posttest lesson-design scores was assessed using the intraclass correlation coefficient (ICC; two-way random-effects model, absolute agreement; ICC(2,1)). The results indicated good agreement between the two raters (ICC = 0.838, 95% CI [0.719, 0.907], F(52, 52) = 6.076, *p* < 0.001, *N* = 53). For subsequent statistical analyses, each participant’s lesson-design performance score was computed as the mean of the two raters’ scores.

#### 4.4.2. Questionnaire Measures

A multi-item questionnaire was administered to assess preservice teachers’ learning-related perceptions and self-regulatory characteristics in the course context. The questionnaire consisted of two sections. The first section collected demographic information. The second section comprised 85 items adapted from established instruments to measure a range of AI-related and learning-related constructs. In the present study, analyses focused on three self-reported learning variables: learning attitude, self-regulated learning (SRL), and critical thinking awareness. All items were rated on a 5-point Likert scale ranging from 1 (strongly disagree) to 5 (strongly agree). To ensure comprehension and measurement accuracy, the questionnaire was administered in Chinese. Translation followed standard translation and back-translation procedures based on Brislin’s guidelines (Brislin, 1970) to ensure linguistic accuracy and conceptual equivalence.

Learning attitude was measured using the Attitudes toward Science Learning scale developed by Hwang et al. (2013). The scale assesses affective perceptions of learning, including interest, perceived value, and engagement. Without altering the original structure or intended meaning, items were contextually adapted to fit the Chemistry Lesson Design course by interpreting references to “science learning” as learning in the present course. The adapted scale consists of seven items (e.g., “I think learning this course is interesting and valuable” and “It is important for me to learn this course well”). The scale showed good internal consistency in the present study (Cronbach’s α = 0.882).

SRL was measured using the Self-Regulated Learning Scale developed by Lee and Tsai (2011). The scale contains seven items reflecting learners’ goal setting, strategy use, self-paced learning, and self-evaluation (e.g., “I can set my own learning goals” and “I can evaluate or review my learning outcomes”). The SRL scale demonstrated good internal consistency (Cronbach’s α = 0.851).

Critical thinking awareness was measured using the scale adapted by Lin et al. (2019), based on the measure proposed by Chai et al. (2015). The scale contains six items capturing learners’ perceived propensity to monitor and evaluate information and evidence during learning activities (e.g., “During the learning process, I judge the value of new information or evidence presented to me” and “I can distinguish which information can be trusted”). The critical thinking awareness scale demonstrated good internal consistency (Cronbach’s α = 0.855).

To provide preliminary evidence of construct validity, exploratory factor analyses were conducted on the pretest items of the three focal subscales. A single-factor structure was supported for learning attitude (KMO = 0.832; Bartlett’s test of sphericity: χ^2^(21) = 192.971, *p* < 0.001), explaining 53.55% of the variance, and for critical thinking awareness (KMO = 0.759; Bartlett’s test of sphericity: χ^2^(15) = 136.547, *p* < 0.001), explaining 50.22% of the variance. The SRL scale was also suitable for factor analysis (KMO = 0.791; Bartlett’s test of sphericity: χ^2^(21) = 185.721, *p* < 0.001), with preliminary results suggesting that its structure should be further examined in larger samples.

### 4.5. Data Analyses

Data analyses were conducted using IBM SPSS Statistics (version 27). Descriptive statistics (means and standard deviations) were calculated for pretest and posttest scores. Baseline equivalence between the two groups was examined using independent-samples *t*-tests on all pretest measures. To examine group differences while controlling for baseline levels, analysis of covariance (ANCOVA) was conducted for each outcome variable, with posttest scores as the dependent variables, instructional condition (AI-FL vs. R-FL) as the fixed factor, and corresponding pretest scores as covariates. Statistical significance was set at *p* < 0.05, and effect sizes were reported using partial eta squared (partial η^2^).

Prior to conducting ANCOVA, the relevant statistical assumptions were examined. The homogeneity of regression slopes was assessed by testing the interaction between instructional condition and pretest scores, and no significant interaction effects were found (all *p* values > 0.05), indicating that the assumption was met. Homogeneity of variances was evaluated using Levene’s test and was found to be acceptable (*p* > 0.05). Normality of residuals was examined and no substantial violations were observed. Data were screened for completeness and response quality prior to inferential analyses; cases with incomplete or low-quality responses were excluded according to predefined criteria.

## 5. Results

### 5.1. Descriptive Statistics

Table 1 presents group-level means and standard deviations for all study variables at pretest and posttest. Descriptive statistics are reported for the experimental and control groups across lesson-design performance, learning attitude, self-regulated learning, and critical thinking awareness.

### 5.2. Baseline Equivalence

Independent-samples *t* tests were conducted to examine baseline equivalence between the experimental and control groups on all pretest measures. As shown in Table 2, no statistically significant between-group differences were found for lesson-design performance, learning attitude, self-regulated learning, or critical thinking awareness prior to the intervention.

### 5.3. Lesson-Design Performance

**Figure 3:**
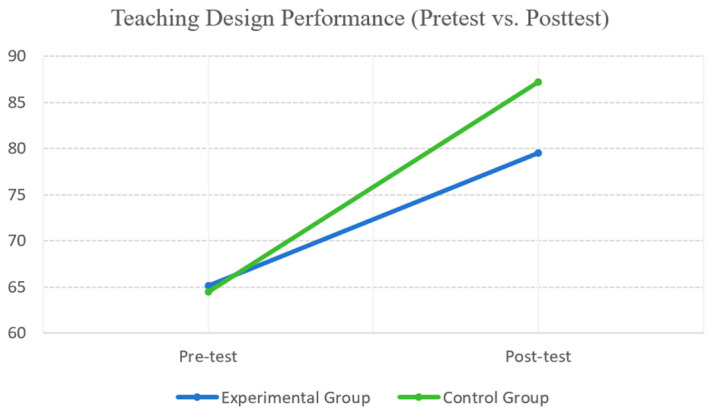
Pretest and posttest lesson-design performance by group. Note. Experimental group = AI-FL (GenAI-supported flipped learning); Control group = R-FL (reading-based flipped learning).

For lesson-design performance, ANCOVA indicated a significant association between pretest and posttest scores, F(1, 50) = 20.809, *p* < 0.001, partial η^2^ = 0.294, suggesting that the pretest score was a meaningful covariate. After controlling for pretest performance, a significant main effect of instructional condition was found, F(1, 50) = 32.791, *p* < 0.001, partial η^2^ = 0.396. The control group achieved higher posttest lesson-design performance than the experimental group (i.e., higher adjusted posttest scores). As shown in Figure 3, both groups improved from pretest to posttest; however, the increase in lesson-design performance was more pronounced in the control group.

### 5.4. Learning Attitude

The ANCOVA results revealed a significant main effect of instructional condition (AI-FL vs. R-FL) on posttest learning attitude after controlling for pretest scores, F(1, 50) = 4.954, *p* = 0.031, partial η^2^ = 0.090. Adjusted posttest scores indicated that the experimental group scored higher than the control group. This pattern suggests a more positive posttest learning attitude under AI-FL after accounting for initial differences.

### 5.5. Self-Regulated Learning

The ANCOVA results indicated no significant main effect of instructional condition (AI-FL vs. R-FL) on posttest self-regulated learning after controlling for pretest scores, F(1, 50) = 0.111, *p* = 0.740, partial η^2^ = 0.002. The covariate (pretest SRL) was significantly related to posttest SRL, F(1, 50) = 9.338, *p* = 0.004, indicating meaningful baseline–posttest association. However, adjusted posttest SRL scores did not differ significantly between the experimental and control groups. This suggests that, under the present implementation, SRL did not differ reliably between the experimental and control groups.

### 5.6. Critical Thinking Awareness

The ANCOVA results indicated that, after controlling for pretest scores, the main effect of instructional condition (AI-FL vs. R-FL) on posttest critical thinking awareness was not statistically significant, F(1, 50) = 3.428, *p* = 0.070, partial η^2^ = 0.064. The covariate (pretest critical thinking awareness) was significant, F(1, 50) = 5.612, *p* = 0.022. Although the adjusted posttest mean was higher in the experimental group than in the control group, this difference did not reach statistical significance. Thus, the evidence did not support a reliable between-group difference in critical thinking awareness in the present study.

## 6. Discussion

With regard to RQ1, a significant between-group difference emerged in objective lesson-design performance, but the direction of this difference was not in line with a simple expectation that GenAI-supported flipped learning would produce superior performance. After controlling for pretest differences, the control group achieved significantly higher posttest lesson-design scores than the experimental group. This finding is closely related to the nature of the outcome examined in this study. Lesson design is a typical criterion-referenced performance task assessed with a rubric that foregrounds principled alignment and internal coherence across instructional objectives, learner analysis, learning activities, and assessment design. As such, it requires sustained integration, reflection, and iterative revision within an authentic design context.

In the present study, reading-based pre-class preparation—curriculum standards, instructional design literature, and exemplary lesson cases—presented core principles and evaluative cues in a relatively stable and traceable manner. This may have helped preservice teachers establish clear evaluative anchors. It may also have supported repeated rubric-based checking and revision during production, thereby increasing the likelihood of stronger posttest performance. This relative advantage of structured reading materials becomes even clearer when contrasted with the kinds of support that GenAI typically provides during pre-class preparation. By contrast, AI-supported pre-class preparation may encourage surface-level textual fluency without a corresponding improvement in rubric alignment. The key issue is not whether GenAI can generate ideas efficiently, but whether learners can adequately verify and calibrate its suggestions. When GenAI outputs display linguistic fluency and seemingly complete structures, learners may converge quickly on a draft that appears plausible. Yet they may invest insufficient effort in rubric-alignment checks and evidence-based verification when transforming it into a final lesson plan—particularly when the workflow does not structurally require cross-checking, counter-checking, and reflective justification (Boud & Molloy, 2013; Selwyn, 2019).

At the same time, large language models still involve risks regarding factuality and inferential reliability. They may present widely held assumptions as facts and increase users’ confidence in inadequately grounded recommendations (Weidinger et al., 2022). Moreover, their outputs often gravitate toward high-frequency generic templates, which can compress the space for contextualised pedagogical reasoning. For lesson-design tasks that depend heavily on fine-grained coherence and standards alignment, even small degrees of inaccuracy, overgeneralisation, or contextual mismatch can trigger cascading misalignment in key dimensions such as learner analysis, activity design, and objective–activity–assessment coherence, thereby lowering rubric scores.

Beyond issues of rubric alignment and verification, cognitive load theory (CLT) offers a complementary explanation for why the AI-supported condition may not have yielded stronger performance in this context. CLT distinguishes intrinsic load, extraneous load, and germane load (Sweller, 1988; Sweller et al., 2011). In AI-supported contexts, outputs can be information-dense and interaction threads can become fragmented. In such cases, the costs of selection and integration increase. Learners may allocate substantial cognitive resources to information-management activities, such as reading, filtering, reorganising, and rewriting. This can increase extraneous load and crowd out working-memory resources needed for principled alignment, justification, and optimisation (Bauer et al., 2023; Moreno, 2004; J. C.-Y. Sun et al., 2019). In addition, less structured presentation can heighten cognitive burden and weaken the functional value of feedback (Kalyuga, 2011). It may also reduce calibrated trust and willingness to verify suggestions (Hudon et al., 2021).

Taken together, under the implementation conditions of this study, reading-based preparation appears more conducive to standards-aligned checking and reflective revision that are tightly coupled with course requirements. In contrast, AI-supported preparation may be more likely to induce reduced evaluative monitoring, premature convergence on generic templates, and increased demands for verification and integration (Carby, 2023; Rob & Rob, 2018). These mechanisms provide a plausible explanation for why the experimental group scored significantly lower than the control group on the posttest lesson-design task.

With regard to RQ2, H2 received partial support. The results showed a differentiated pattern: the AI-supported condition was associated with higher learning attitude, whereas no significant differences were found for self-regulated learning, and the effect on critical thinking awareness was marginal. This divergence suggests that GenAI-supported flipped learning may more readily produce motivational and engagement-related benefits through immediacy and interactivity. By contrast, changes in self-regulatory and evaluative capacities may be more contingent on how the intervention is structurally scaffolded and enacted in practice (Selwyn, 2019; Sweller, 1988; Sweller et al., 2011).

Regarding learning attitude, the higher learning attitude may be attributed to the immediacy of GenAI responses and students’ perceptions of responsive support during interaction. Such responsiveness can strengthen perceived usefulness and reduce frustration during pre-class preparation, thereby increasing willingness to invest effort. In feedback research, supportive language and responsive communication enhance learners’ engagement and receptiveness to guidance (Hyland & Hyland, 2006). Learners also tend to prefer teacher feedback or blended sources when they perceive relational credibility and interpretive clarity (Zeevy-Solovey, 2024). In this sense, GenAI may offer readily available, immediate assistance that supports students’ motivation, even if it cannot fully replicate the dialogic and contextual qualities of human guidance (Boud & Molloy, 2013). This suggests that affective–motivational benefits may emerge earlier than measurable gains in complex professional performance. However, because posttest learning attitude scores were relatively high in both groups, a possible ceiling effect cannot be ruled out. This may have reduced the measure’s ability to capture more subtle between-group differences, and the finding should therefore be interpreted with caution.

Regarding self-regulated learning (SRL), the non-significant result is not surprising. SRL is a relatively stable competence and typically requires sustained, explicit scaffolding and repeated practice rather than short-term exposure to a supportive tool. Moreover, SRL development depends on accountability structures. Without routines that externalise planning, monitoring, and reflection, learners may appropriate GenAI in efficiency-oriented ways (e.g., obtaining quick suggestions) rather than practising regulatory procedures. Socio-constructivist perspectives further emphasise that what matters is not only information provision but also dialogic clarification and feedback dialogue, which are central to developing reflective routines (Boud & Molloy, 2013). The implication is that, to promote SRL in AI-FL, it is necessary to embed structural scaffolds (e.g., planning templates, monitoring checklists, reflection prompts, and evaluation checkpoints) that make regulation visible and practiced rather than assumed.

Regarding critical thinking awareness, the marginal effect may partly reflect the self-report nature of the measure. It may also relate to the epistemic demands involved in evaluating GenAI outputs. When learners perceive AI suggestions as fluent yet insufficiently transparent or context-attuned, they may either distrust the output or over-rely on surface plausibility—both of which can constrain reflective monitoring (Selwyn, 2019). Empirical discussions of AI feedback note challenges in empathy and contextual understanding, which can lead to suboptimal outcomes when learners experience feedback as mechanistic or misaligned (Wang et al., 2024). Skepticism about transparency and reliability further shapes adoption behavior and credibility judgments (Selwyn, 2019; Zhang et al., 2025). The implication is that critical thinking awareness may require designs that make evaluation unavoidable (e.g., evidence checks, counter-argument prompts, and source comparison tasks), rather than assuming that access to GenAI will automatically cultivate evaluative habits.

Across SRL and critical thinking awareness, CLT provides a unifying explanation: when interaction generates dense and fragmented outputs, extraneous load rises, leaving fewer resources for evaluation, justification, and reflective monitoring (Kalyuga, 2011; Moreno, 2004; J. C.-Y. Sun et al., 2019; Sweller, 1988; Sweller et al., 2011). This helps explain why GenAI support can elevate learning attitude (a relatively faster-moving perception) while yielding limited short-term changes in SRL and only marginal gains in critical thinking awareness (Hudon et al., 2021).

## 7. Conclusions and Implications

This quasi-experimental study compared GenAI-supported flipped learning (AI-FL) and reading-based flipped learning (R-FL) in a preservice chemistry teacher education course. Overall, the hypotheses were only partially supported. A significant between-group difference emerged in lesson-design performance, but the direction of the difference favored the control group rather than the experimental group. The hypothesis regarding subjective learning perceptions received partial support. Compared with the control group, the experimental group showed higher posttest learning attitude (adjusted for pretest). By contrast, no significant between-group difference was found for self-regulated learning, and only a marginal effect was observed for critical thinking awareness.

Taken together, the results suggest that, for rubric-scored lesson-design production tasks, access to GenAI alone is unlikely to yield reliable improvements in product quality. Better performance is more plausible when the learning design embeds standards-aligned checking, evidence-based verification, and iterative revision as required workflow components—especially when these processes carry explicit assessment consequences. From this perspective, what matters in lesson-design courses is not simply whether GenAI is used, but how its use is pedagogically structured and constrained.

In lesson-design courses, GenAI should be positioned as a constrained decision-support resource rather than an open-ended drafting tool.

In the pre-class phase, the task sheet can require a rubric-linked alignment artefact prior to drafting (e.g., an objectives–activities–assessment alignment matrix mapped to rubric dimensions). Students can also submit a brief verification note indicating which curriculum-standard clauses, exemplar excerpts, or design principles support each key instructional decision.

In the in-class phase, calibration can be organised using rubric language. For example, students can be required to identify at least two rubric-specific misalignments in their draft, implement targeted revisions, and justify the revisions explicitly in terms of rubric criteria rather than focusing primarily on stylistic refinement.

At the assessment level, verification and calibration can be made consequential by adding a process-oriented scoring dimension. Alternatively, a mandatory graded appendix can document standards- or case-based warrants, revision evidence, and rubric-grounded revision rationales, so that alignment checking and iterative revision become required elements of task completion.

Finally, GenAI-use guidance should explicitly manage risks of generic framing and overconfident adoption in educational production tasks. Prompt constraints can require students to elicit alternatives with explicit trade-offs, articulate assumptions and plausible failure points, and specify how solutions should change under different learner profiles. These steps can then be followed by rubric-based selection and justification. Brief AI-literacy micro-tasks can also be embedded (e.g., flagging suggestions requiring verification, identifying potential bias or overgeneralisation, and cross-checking at least one key claim against standards or exemplars) so that reliability and evidential sufficiency checks become routine during production.

Several limitations should be considered. First, this study compared two pre-existing intact classes rather than using individual random assignment. Although baseline equivalence was examined and the two groups were taught by the same instructor under the same syllabus, timeline, and assessment criteria, the observed intervention effect cannot be cleanly separated from pre-existing differences between the two classes. Therefore, strong causal inferences should be avoided. Second, although the instructor specified the preview themes, provided guiding questions, and offered session-by-session guidance on AI interaction, the specific GenAI platforms and settings were not fully standardized in the AI-FL condition, which may have introduced some tool-related variability. In addition, although students in the R-FL condition were explicitly instructed to complete pre-class preparation through the assigned readings rather than AI-based tools, and this requirement was reiterated before each weekly task, it was not possible to fully verify compliance because the pre-class phase took place outside the classroom. Third, the sample size and the single-course context may limit the generalisability of the findings to other institutions, disciplines, or cultural settings. The relatively small sample also limited the extent to which the factorial validity of the adapted self-report measures could be thoroughly examined in the present study. Fourth, the relatively short implementation period and reliance on self-report measures may have reduced sensitivity to short-term change in relatively stable constructs such as self-regulated learning and critical thinking awareness. In addition, because posttest learning attitude scores were relatively high in both groups, a possible ceiling effect cannot be ruled out. This measurement-related constraint may have reduced the sensitivity of the scale to more subtle between-group variation on this outcome.

Future research may replicate this comparison with larger, multi-site samples and, where feasible, adopt randomised or crossover designs. More controlled pre-class procedures may also be introduced to better monitor compliance with group-specific learning requirements. One possible extension would be to include a third condition in which students complete assigned readings and use GenAI for specific instructional purposes. This design would allow a more fine-grained comparison of reading-only, AI-supported, and reading-plus-AI pre-class preparation, and help clarify whether GenAI functions more effectively as a substitute for, or as a supplement to, structured reading in flipped learning. Richer process evidence, such as GenAI interaction logs, revision histories, and rubric-alignment trace data, should also be collected to examine whether rubric coupling and embedded verification checkpoints moderate the effects of GenAI-supported flipped learning on lesson-design outcomes. Finally, future studies should further examine the factorial validity of the adapted self-report measures using larger samples.

## Figures and Tables

**Table 1:** Descriptive statistics for study variables at pretest and posttest by group.

Variable	Group	Pretest, M (SD)	Posttest, M (SD)
Lesson-design performance	Experimental	65.17 (10.06)	79.50 (6.08)
	Control	64.50 (7.08)	87.24 (5.85)
Learning attitude	Experimental	4.08 (0.39)	4.72 (0.39)
	Control	4.18 (0.50)	4.52 (0.43)
Self-regulated learning	Experimental	4.12 (0.40)	4.27 (0.34)
	Control	4.03 (0.37)	4.27 (0.52)
Critical thinking awareness	Experimental	4.00 (0.47)	4.22 (0.36)
	Control	4.10 (0.49)	4.05 (0.46)

Note. Experimental group = AI-FL (GenAI-supported flipped learning); Control group = R-FL (reading-based flipped learning).

**Table 2:** Baseline equivalence between the experimental and control groups on pretest measures.

Variable	Experimental, M (SD)	Control, M (SD)	t	df	*p*
Lesson-design performance	65.17 (10.06)	64.50 (7.08)	0.282	51	0.779
Learning attitude	4.08 (0.39)	4.18 (0.50)	−0.750	51	0.457
Self-regulated learning	4.12 (0.40)	4.03 (0.37)	0.804	51	0.425
Critical thinking awareness	4.00 (0.47)	4.10 (0.49)	−0.779	51	0.439

Note. Experimental group = AI-FL (GenAI-supported flipped learning); Control group = R-FL (reading-based flipped learning).

## Data Availability

The datasets used and/or analysed during the current study are available from the corresponding author on reasonable request.

## Abbreviations

The following abbreviations are used in this manuscript:

GenAI	generative artificial intelligence
AI-FL	GenAI-supported flipped learning
R-FL	reading-based flipped learning
FL	flipped learning
SRL	self-regulated learning
ANCOVA	analysis of covariance
ICC	intraclass correlation coefficient
CLT	cognitive load theory

## Appendix A. Overview of the 11-Week Intervention: Weekly Topics and Guiding Questions

To improve the transparency and reproducibility of the intervention, Appendix A summarises the 11-week course structure, including the weekly focus and the instructor-provided guiding questions used during the nine guided-question instructional sessions. These questions served as a common scaffold for pre-class preparation in both conditions. In the AI-FL condition, students used them to guide GenAI-supported inquiry; in the R-FL condition, students used them to guide reading-based preparation.

**Table A1:** Weekly structure, topics, and instructor-provided guiding questions in the 11-week intervention.

Week	Phase/Purpose	Topic	Instructor-Provided Guiding Questions
1	Orientation and pretest	Course introduction and study orientation	—
2	Guided-question instructional session	Introduction to chemistry instructional design	(1) What is instructional design, and what are the main components of a complete instructional design? (2) What characteristics should a high-quality chemistry instructional design possess? (3) Identify an example of an excellent chemistry instructional design, analyse its main strengths, and suggest possible improvements. (4) How can instructional design be developed for a specific chemistry teaching topic?
3		Senior secondary chemistry curriculum standards and core competencies	(1) What are the main contents of the senior secondary chemistry curriculum standards? (2) How should the core competencies of chemistry be understood? (3) How should the implementation recommendations in the chemistry curriculum standards be interpreted?
4		Design of teaching objectives	(1) What role do teaching objectives play in instruction? (2) What principles should be followed in designing teaching objectives? (3) What methods can be used to design chemistry teaching objectives?
5		Design of teaching situations	(1) What is the theoretical basis for creating teaching situations in chemistry instruction? (2) What are the main types of teaching situations in chemistry teaching? (3) How can appropriate teaching situations be created in chemistry instruction?
6		Selection of teaching methods	(1) What are the common teaching methods used in chemistry instruction? (2) What factors influence the selection of teaching methods? (3) How can appropriate teaching methods be selected in chemistry teaching?
7		Design of teaching activities	(1) What is the theoretical basis for designing chemistry teaching activities? (2) What are the main types of chemistry teaching activities? (3) What general principles should be followed in designing chemistry teaching activities?
8		Design of teaching media	(1) What is the theoretical basis for chemistry teaching media design? (2) What are the main types of teaching media used in chemistry instruction? (3) What strategies can be used for chemistry teaching media design in the digital era?
9		Design of board writing	(1) What are the theoretical basis and instructional value of board writing? (2) What are the main types of board writing used in chemistry teaching? (3) How can board writing be effectively designed in chemistry instruction?
10		Teaching evaluation	(1) What are the theoretical basis and instructional value of teaching evaluation? (2) What are the main types of teaching evaluation? (3) How can teaching evaluation be effectively implemented in chemistry instruction?
11	Final task and posttest	Comprehensive lesson-design task and posttest	—

## Appendix B. Lesson-Design Evaluation Rubric

To improve the transparency and reproducibility of the objective performance assessment, the lesson-design evaluation rubric used in this study is provided in Table A2. The rubric was adapted from the official evaluation criteria of the Tian Jiabing Cup National Teaching Skills Competition and was used to assess participants’ lesson-design performance. The total possible score of the original rubric was 25. In this study, the original 25-point rubric score was linearly rescaled to a 100-point scale for consistency with the course assessment system; however, Table A2 presents the original rubric structure and scoring scheme.

**Table A2:** Lesson-design evaluation rubric adapted from the official evaluation criteria of the Tian Jiabing Cup National Teaching Skills Competition.

Dimension	Criterion	Score
Objective design	(1) Objectives are clear, specific, understandable, and feasible; action verbs are used appropriately and wording is standardized.	1.5
	(2) Objectives align with curriculum standards, reflect disciplinary features and student needs, and address knowledge, abilities, and innovative thinking.	1.5
Content analysis	Relationships among prior, current, and subsequent knowledge are accurately described; key and difficult points are clearly identified.	2
Learner analysis	Students’ cognitive characteristics, prior knowledge, learning habits, and abilities are appropriately analyzed.	2
Teaching process design	(1) The instructional sequence is clear, coherent, and logically organized, and content treatment aligns with curriculum standards.	2
	(2) Key points are emphasized, links are well integrated, depth is appropriate, and difficult points are accurately addressed.	2
	(3) Teaching methods are appropriate for learner characteristics and support task completion, key-point emphasis, and difficulty resolution.	2
	(4) Teaching aids, instructional materials, and modern technologies are adequately prepared and appropriately used.	1
	(5) Content is substantial and suitable for students’ level; the structure is well organized, practical, interactive, and conducive to thinking and problem solving.	3
	(6) Formative assessment is emphasized, together with the generation, resolution, and use of meaningful instructional problems.	1
Extension design	Time allocation and support activities are appropriate; exercises, assignments, and discussions align with objectives and promote understanding and problem solving.	2
Document quality	Text, symbols, units, and formulas are standardized; language, layout, and formatting are clear, complete, and appropriate.	2
Design innovation	The lesson-plan design is innovative and reflects curriculum reform principles.	3
Total		25
